# Molecular survey of *mcr1* and *mcr2* plasmid mediated colistin resistance genes in *Escherichia coli* isolates of animal origin in Iran

**DOI:** 10.1186/s13104-021-05519-6

**Published:** 2021-03-23

**Authors:** Kayhan Ilbeigi, Mahdi Askari Badouei, Hossein Vaezi, Hassan Zaheri, Sina Aghasharif, Khatereh Kafshdouzan

**Affiliations:** 1 Laboratory of Microbiology, Parasitology and Hygiene (LMPH), Department of Biomedical Sciences, University of Antwerp, Wilrijk, Belgium; 2 Department of Pathobiology, Faculty of Veterinary Medicine, Garmsar Branch, Islamic Azad University, Semnan, Iran; 3 Department of Pathobiology, Faculty of Veterinary Medicine, Semnan University, Semnan, Iran

**Keywords:** Colistin resistance, Food-producing animals, *mcr-1*, *mcr-2*

## Abstract

**Objectives:**

The emergence of colistin-resistant *Enterobacteriaceae* from human and animal sources is one of the major public health concerns as colistin is the last-resort antibiotic for treating infections caused by multidrug-resistant Gram-negative bacteria. We aimed to determine the prevalence of the prototype widespread colistin resistance genes (*mcr-1* and *mcr-2*) among commensal and pathogenic *Escherichia coli* strains isolated from food-producing and companion animals in Iran.

**Results:**

A total of 607 *E. coli* isolates which were previously collected from different animal sources between 2008 and 2016 used to uncover the possible presence of plasmid-mediated colistin resistance genes (*mcr-1* and *mcr-2*) by PCR. Overall, our results could not confirm the presence of any *mcr-1* or *mcr-2* positive *E. coli* among the studied isolates. It is concluded that despite the important role of food-producing animals in transferring the antibiotic resistance, they were not the main source for carriage of *mcr-1* and *mcr-2* in Iran until 2016. This study suggests that the other *mcr* variants (*mcr-3* to *mcr-9*) might be responsible for conferring colistin resistance in animal isolates in Iran. The possible linkage between pig farming industry and high level of *mcr* carriage in some countries needs to be clarified in future prospective studies.

## Introduction

Since the advent of antimicrobial agents for clinical practices, researchers have been faced with the emergence and spread of resistant bacteria. Mechanisms of resistance are known to favor microbial growth even in high concentration of therapeutics, causing treatment failures. This phenomenon is continuously being explored in both human and veterinary medicine. The agricultural use of antibiotics is increasing, either as growth promoters or as therapeutic agents in farm animals [1]. This could be potentially a threat to human health because resistant organisms developed in food-producing animals may confer resistance to human commensal or pathogenic strains through the food chain or direct contacts [1, 2].

Colistin is an antibiotic belonging to the polymyxins family. This drug is among the top most critically important antimicrobial agents in veterinary medicine and is active against a broad range of Gram-negative bacteria including most members of the *Entrobacteriaceae* family [3]. Colistin targets the polyanionic lipid A of lipopolysaccharides in the outer membrane of Gram-negative bacteria [4]. This antibiotic is widely used in poultry farms for the prevention and treatment of *Enterobacteriaceae* infections and is the last-resort antibiotic for treating multidrug-resistant (MDR) infections [5]. Microbial resistance to colistin occurs mostly among isolates from food animals compared to the human isolates [6–10]. This is due to the continuous use of colistin in veterinary medicine which has led to the higher rates of colistin resistance among isolates from food animals [8]. Moreover, the emergence of extensive drug-resistant (XDR) Gram-negative bacteria (GNB) has further increased the interest of most scientists to use colistin for the treatment of severe infections caused by these pathogens [11]. Most polymyxin resistance mechanisms are chromosomally mediated and involve modulation of a two-component regulatory system (*e.g*., *pmrAB*, *phoPQ*, and the negative regulator *mgrB*), leading to modification of lipid A with moieties including phosphoethanolamine, 4-amino-4-deoxy-l-arabinose, or, in rare instances, the total loss of lipopolysaccharides [7, 12]. Recently, it was discovered that the *mcr-1* (mobile-colistin-resistance) gene is associated with colistin resistance. This gene codes for enzymes capable of modifying the LPS through the addition of phosphoethanolamine [7]. The emergence of the *mcr-1* gene could be a potential threat to the efficacy of polymixins and is a global concern. This gene has been detected in various *Enterobacteriaceae* species from the environment, food, humans, livestock, wildlife, companion animals, rivers, and vegetables in more than 50 countries and regions [13, 14]. Reports have shown evidence of eight other plasmid-mediated colistin resistance genes which include *mcr-2* to *mcr-9* [15]. Although colistin has been frequently used in veterinary medicine particularly in poultry in Iran, the prevalence of colistin resistance and the carriage of *mcr* genes among the commensal enteric bacterial isolates from food-producing animals is poorly understood. This study was conducted to survey the frequency of the prototype widespread colistin resistance genes (*mcr-1* and *mcr-2*) among commensal and some pathogenic enteric bacteria (*E. coli*) isolated from animals in Iran.

## Main text

### Material and methods

A total of 607 *E. coli* isolates were collected from different animal sources including broilers, ostriches, cattle, and sheep, pigeons and dogs. These bacteria were isolated from various regions of Iran from 2008 to 2016 in the context of different previous studies. The number of samples from each animal species and geographical regions are presented in Table 1. Briefly, the original sampling procedure included collecting fecal samples using sterile cotton swabs from the rectum of cattle, sheep and dogs, and fresh droppings of pigeons and ostriches. For the extraintestinal pathogenic *E. coli* strains, the samples were aseptically taken from bovine mastitis milk and liver or heart blood of commercial broilers recently died of colibacillosis. The Shiga-toxin producing *E. coli* (STEC) strains were confirmed by different molecular methods described previously [16]. All animals were belonging to commercial production systems except dogs and pigeons. In these cases, samples were only obtained upon the owners’ consent and approval. The samples were streaked on MacConkey agar (Merck, Germany) and a pure isolate from each sample was confirmed as *E. coli* using standard biochemical tests [17]. All isolates were cryopreserved in Brain Heart Infusion broth (Merck, Germany) with 30% glycerol as stocks and kept at − 70 °C. The archived strains were recovered on Brain Heart Infusion broth (BHI; Merck, Germany) after overnight culture at 37 °C with an additional streak on MacConkey agar to confirm the purity of the stocks, and a single pure colony was assessed by molecular methods; when necessary, the identity of the isolates was reassessed using sets of standard biochemical tests as described in laboratory manuals. The tests included using differential media such as Triple Sugar Iron agar, Urea agar, SIM, Citrate, and MR-VP (Merck, Germany) [17]. *E. coli* carrying *mcr-1* (2012-60-1176-27) and *E. coli* containing *mcr-2* (KP37) were used as reference strains and positive controls in PCR reactions. All strains were tested by two sets of conventional PCR assays targeting the *mcr-1* and *mcr-2* resistance genes. DNA templates were extracted by boiling method as described previously [18]. Briefly, a loopful from an overnight culture on Luria Bertani agar (Merck, Germany) was suspended in 350 μl molecular grade water. Then, the suspension was boiled for 10 min and after cooling on ice, centrifuged for 5 min at 10,000×*g* and the supernatants were used as templates. For PCR reactions, a ready-to-use Red PCR Master Mix (Ampliqon, Denmark) containing 1.5 mM MgCl_2_, 0.2 mM dNTP and 0.1% Tween 20 was used. The final concentration for each primer was 0.3 μM, and 3 μl of crude DNA was used as template. The primers were (5′-CGGTCAGTCCGTTTGTTC -3′/5′-CTTGGTCGGTCTGTAGGG-3′) for *mcr-1,* and (5′-TGTTGCTTGTGCCGATTGGA -3′/5′-AGATGGTATTGTTGGTTGCTG-3′) for *mcr-2* as described before [7, 19]. Thermal cycling conditions were conducted in TC-3000 cycler (Techne, UK) under the following conditions: for the *mcr-1* amplification, one cycle of denaturation at 94 °C for 5 min, followed by 30 cycles of denaturation at 94 °C for 30 s, annealing at 58 °C for 90 s and elongation at 72 °C for 60 s, and a final cycle of elongation at 72 °C for 7 min was applied. The amplification of *mcr-2* included 33 cycles of 95, 65 and 72 °C, all for 60 s. The amplification output was visualized by electrophoresis on 1.5% agarose gel at 100 V followed by staining in DNA safe stain (Cinnagen, Iran).

**Table 1:** The origin and characteristics of the 607 studied *E. coli* isolates

Animal origin	Province(s)	Year of isolation	Pathotype/commensal (Number)
Broiler chickens	Semnan	2014–2016	APEC^a^ (183)
Ostrich	Semnan Gilan Yazd	2012–2016	Fecal (70) Septicemic (35)
Pigeon	Tehran Semnan Khorasan Mazandaran	2012–2014	STEC^b^ (33)
Bovine Mastitis	Semnan Tehran	2012–2015	MPEC^c^ (36)
Cattle	Tehran Semnan Golestan Khorasan	2008–2016	STEC (94)
Sheep	Semnan Khuzestan Kerman Fars	2013–2016	STEC (51) Commensal (31)
Dog	Semnan Tehran	2010–2011	Commensal (74)

^a^ Avian pathogenic *E. coli*

^b^ Shiga toxin-producing *E. coli*

^c^ Mammary pathogenic *E. coli*

### Results and discussion

In the present survey, no *mcr-1*/*mcr-2* harboring *E. coli* were found among the 607 tested isolates of animal origin which belonged to ten provinces in Iran. Antibiotic consumption or administration in animals is known as a major contributor to the development of antimicrobial resistance in humans. The recent emergence of clinically important bacteria such as *mcr*-positive colistin-resistant *E. coli* in humans has been mostly associated with food-producing animals [20]. Globally, colistin is among the widely used antibiotics in veterinary clinical practices. The *mcr* genes associated with colistin resistance in *Enterobacteriaceae* are widespread and have been reported in veal, swine, and poultry in different countries [7, 21–23]. The aforesaid resistance has already become established in livestock, posing a potential threat to consumers through eating contaminated meat and other products [24].

Generally, few studies have investigated colistin resistance by molecular methods in *E. coli* isolates from animals in Iran. Based on the CLSI guidelines, the disc diffusion method cannot reflect resistance to colistin or harboring mobile genetic *mcr* elements, suggesting that some of the previous colistin susceptibility studies in Iran could be misleading. In this study, a PCR-based detection method was performed to survey the *mcr-1* and *mcr-2* presence among an extensive collection of *E. coli* strains, and none of the tested isolates carried the targeted genes. As mentioned, these strains were recovered from 2008 to 2016 from different animal sources. In a similar study, no colistin-resistant *E. coli* isolate was detected in poultry in the northwest of Iran [25]. Another study from southwest Iran reported 1.2% of *E. coli* and 0.4% of *K. pneumoniae* isolates from human samples carried *mcr-1* gene while *mcr-2* was not detected [26]. Consistently, in a recent study, no *mcr-1* and *mcr-2* genes were identified from *Enterobacteriaceae* isolates of human origin in the northwest of Iran [27]. Despite the regular administration of colistin in farm animals, the absence of *mcr-1 and mcr-2* genes suggested that these genes were not widespread among animal sources in Iran at least until few years ago. Additionally, these findings partially support the low prevalence of *mcr* genes in human and veterinary clinical isolates in previous studies conducted in Iran.

The *mcr-1,* as a prototype of the plasmid resistance gene for colistin, was initially reported in *E. coli* from livestock, food, and humans in China in 2015 [28]. A study in China described a low prevalence of colistin-resistance *E. coli* among cattle (0.9%); while in comparison, such resistance among *E. coli* isolated from chickens and pigs was high (14% among chickens and 24% among pigs) [10]. The gene was also reported from Denmark and other countries confirming worldwide dissemination of the gene from various sources [23, 29]. A low prevalence of *mcr-1* (1%) was detected in *Salmonella* from poultry meat (K. Veldman et al., unpublished data), *E. coli* isolates from livestock (1%) and meat (2%) in the Netherlands [29]. In Germany, a recent study investigating a total of 580 *E. coli* isolates from chicken meat, indicated a decreasing prevalence of *mcr-1*, from 8.1% in 2011 to 0.5% in 2014 based on isolate screening [30]. At much lower rates, the presence of *mcr-1* was confirmed in isolates from poultry and other meat products from Europe in other studies [29]. The *mcr-1*-harboring *E. coli* isolates (19.5%) in chicken meat was also reported from South America based on a selective culture approach [31]. Similar reports demonstrated the presence of *mcr-1* in an *E. coli* isolate from a cow displaying subclinical mastitis, suggesting that *mcr* associated resistance also emerged in Egypt despite showing a very low prevalence in this country [32]. After reporting the first *mcr-1*, the *mcr-2* plasmid-mediated colistin resistance was subsequently described in *E. coli* from cattle and pigs in Belgium [19]. In China, the prevalence of *mcr-2* in colistin-resistant *E. coli* isolates from pigs, chickens and cattle was 46.82%, 14.90%, and 19.05%, respectively, compared to the higher prevalence of *mcr-1* in these species [33]. Other studies reported no *mcr-2* in *E. coli* isolates from cattle, swine, and broilers, while the prevalence of *mcr-1* slightly increased [9, 34]. Additionally, the *mcr-2* was not detected in any poultry isolates in Romania [35].

It seems that the pig industry may play an important role in colistin resistance emergence and spread. In Portugal, 98% of pigs tested positive for *mcr-1* harboring *Enterobacteriaceae* isolates (mainly *E. coli*), while no *mcr-2* carrying isolate was identified [36]. Accordingly, a recent study on the prevalence of colistin-resistant bacteria in Ecuador showed that 41.9% of *E. coli* isolates from chicken and pigs harbored *mcr-1* [37]. Another study in Spain revealed that the prevalence of colistin-resistant *E. coli* was 76.9% in pigs [15]. Also, a high prevalence of *E. coli* harboring *mcr* genes in pigs was detected in China (*mcr-1* = 79.2%, *mcr-2* = 56.3%) [38]. Other studies described the occurrence of *mcr-1* in swine; for example, the prevalence in Europe ranged from 0.5 to 13.5% [36]. Therefore, based on the available literature, pigs have been an important reservoir of colistin plasmid-mediated resistance, and the absence of swine industrial farming in Iran or other countries like Egypt could be a possible reason for the low prevalence of *mcr-1* and *mcr-2* genes in food animals. It should be noted that many novel *mcr* variants need to be targeted in future studies in Iran to obtain a more integrated view on colistin resistance. Also, the presence of possible new *mcr* variants in Iran should be considered.

In conclusion, the results of this study indicated that despite the important role of animals in transferring antimicrobial resistance, we need to have a more integrated perspective about this global issue [39–41]. Additionally, in order to reduce the probability of transmitting resistant bacteria from animals to humans, the monitoring and antibiotic stewardship strategies should be improved.

## Limitations

As we described in Table 1, the studied samples in this survey originated from ten provinces in Iran, but we still need to carry out more sophisticated comprehensive studies to confirm the absence of *mcr-1* and *mcr-2* colistin resistance in the animal population.

## Data Availability

The datasets used and/or analyzed during the current study are available from the corresponding author on reasonable request.

## Abbreviations

MDR: Multidrug-resistant
XDR: Extensive drug-resistant
GNB: Gram-negative bacteria
BHI: Brain heart infusion agar
WGS: Whole genome sequencing
